# Effects of neprilysin-renin inhibition in comparison with neprilysin-angiotensin inhibition on the neurohumoral changes in rats with heart failure

**DOI:** 10.1186/s40360-019-0304-z

**Published:** 2019-05-03

**Authors:** Kawa Dizaye, Rojgar H. Ali

**Affiliations:** 1 College of Medicine, Hawler Medical University, Minara village A05, Erbil, Iraq; 2 College of pharmacy, Hawler Medical University, Erbil, Iraq

**Keywords:** Neprilysin, Sacubitril, Neurohumoral changes, Heart failure, Angiotensin inhibitors

## Abstract

**Background:**

The activation of neurohumoral compensatory mechanisms is a common physiological phenomenon in heart failure in order to make up for a failing heart, which will usually have a deteriorating effect on overall health condition. Many medications, such as neprilysin and angiotensin inhibitors, have recently been introduced to remediate neurohumoral changes. This study was conducted to evaluate the efficacy of the sacubitril-aliskiren combination versus the sacubitril-ramipril combination in the treatment of neurohumoral changes in rats with experimentally induced heart failure.

**Method:**

Thirty Wister rats were randomly assigned into five groups each of six rats, the first group was the control group. Intraperitoneal isoprenaline injections of 5 mg/kg/day for 1 week were used to induce experimental models of heart failure in rats of the rest of experimental groups. The second group served as a positive control. Rats in the third, fourth, and fifth groups received oral daily dose of sacubitril 30 mg/kg/day, sacubitril-aliskiren 30,10 mg/kg/day, and sacubitril-ramipril 30/10 mg/kg/day respectively, for 2 weeks.

**Results:**

Induction of heart failure in rats has significantly increased circulating NT-proBNP (980 ± 116.71 pg/ml), MMP9 (15.85 ± 0.57 ng/ml), troponin-I (3.09 ± 0.147 ng/ml), CK-MB (31.55 ± 1.69 ng/ml), renin (736 ± 45.8 pg/ml), urea (52.1 ± 1.57 mg/dl), and creatinine (0.92 ± 0.04 mg/dl). Significant decreases in glomerular filtration rate (7.031 ± 1.6 ml/hr./kg), urine flow (0.2761 ± 0.06 ml/h/kg), total solute excretion (0.11 ± 0.03 meq/m), and mean blood pressure (83.5 ± 2.6 mm hg) were seen in rats with heart failure.

Rats treated with sacubitril combined with aliskiren or ramipril showed a statistically significant reduction of NT-proBNP, MMP9, troponin serum urea, and serum creatinine.

Sacubitril-aliskiren or sacubitril-ramipril administration produced a significant increase in renin plasma level, total solute excretion, urine flow, and glomerular filtration rate.

**Conclusion:**

Sacubitril in combination with aliskiren or with ramipril effectively reduced plasma cardiac biomarkers, such as CK-MB, MMP9, and NT-proBNP, in rats with heart failure. Both combinations showed significant remediation of renal function through increasing GFR, urine flow, and total solute excretion, as well as reducing plasma level of renin. Net results revealed that the sacubitril-aliskiren combination has similar remediating effects on neurohumoral changes compared to the sacubitril-ramipril combination.

## Background

Heart failure is a pathological condition that occurs when heart is not able to pump sufficient blood to meet physiological requirements, which may lead to many complications like edema, shortness of breath, and possibly death [1, 2].

Physiological changes associated with the heart promote vasoconstriction and enhancing blood flow to confer sufficient ventricular filling. A failing heart is usually associated with neurohumoral changes; when under normal physiological function, the changes make up for the extra load on cardiac walls, but when prolonged, they could play a critical role in the deterioration of overall health. Neurohumoral changes include enhancing the renin-angiotensin-aldosterone system (RAAS), which in turn leads to increased concentrations of plasma renin, angiotensin II, and Aldosterone. Aldosterone increases water and sodium reabsorption and enhances the excretion of potassium. Angiotensin II stimulates the release of noradrenaline from sympathetic nerve terminals and promotes the release of Aldosterone and vasoconstriction. These actions lead to the retention of sodium and water and the increased excretion of potassium [3].

Increased stress on cardiac myocyte will trigger the release of natriuretic peptides (NPs). NPs are a family of hormones that help to maintain sodium and fluid balance though promoting natriuresis and vasodilation. Three NPs have been identified: ANP, BNP, and CNP. ANP is primarily released from the cardiac atrium in response to increased atrial pressure. BNP is released mainly from the left ventricle as a result of ventricular wall stretch [4].

The main physiological actions of NPs are enhancing sodium-water excretion; relaxing the vascular smooth muscle; and reducing or inhibiting the release of endothelin, aldosterone, angiotensin II, and antidiuretic hormone [5]. One of the main limitations of their clinical applications is their short half-life, which is around 4 min for the ANP type and 40 min for BNP type, as these peptides are quickly cleared by an enzyme known as neutral endopeptidase, or neprilysin [3].

Neprilysin is expressed in several tissues but most commonly in the kidney. It terminates the action of many endogenous substances, such as bradykinin, NPs, angiotensin II, and substance P [6].

Diuretics, angiotensin-converting enzyme inhibitors, angiotensin receptor blockers, and β-adrenoreceptor blockers are the main therapeutic agents for the management of heart failure [6].

In the past decade, in the search to improve the management of heart failure, neprilysin inhibitors—such as candoxatril, sacubitril, and omapatrilat—were introduced. Few studies are available to check the efficacy of these medications to alleviate neurohumoral changes experimentally [7].

Since using neprilysin inhibitor alone will increase angiotensin II level, therefore, combining sacubitril with either ACE inhibitors or renin inhibitors could provide further relief of neurohumoral changes associated with heart failure [8].

This study was aimed to evaluate the effect of the neprilysin inhibitor sacubitril in combination with ramipril versus its combination with aliskiren on neurohumoral changes in the treatment of rats with isoprenaline-induced heart failure.

## Methods

### Animals

Thirty female Wistar albino rats weighing 200–240 g were used in this study. The animals were purchased from Zakho Center for Experimental Animals (Iraq, Duhok). Rats were kept in special cages in the animal house of the College of Medicine at Hawler Medical University (Iraq, Erbil). They had free access to water and standard rat-pellet food. The animal room was set on 12/12 h of light-dark cycles.

### Materials

Serum matrix metalloproteinase 9 (MMP9), N-terminal pro B-type natriuretic peptide (NT-proBNP), renin, creatine kinase-MB (CK-MB), and troponin-I Enzyme Linked Immunosorbent Assay (ELISA) rat kits were purchased from Elabsciences (Houston, Texas, 77079, USA).

Isoprenaline HCl was purchased from creative enzymes (Shirley, New York, 11,967, USA), aliskiren 300 mg tablet and ramipril 10 mg tablet were purchased in a verified and licensed pharmacy manufactured by Actavis (Devonshire, UK) and Novartis (Basel, Switzerland) respectively. Starch was used as a placebo in this study; sacubitril calcium (CAT#: 314262, AHU377) was purchased from MedKoo Biosciences, Inc. (Morrisville, North Carolina, 27560, USA).

### Study design

In this study, 30 female Wister albino rats weighing (200–285) g (5 to 6 months old) were allocated into five groups with simple random sampling method (*n* = 6):

- Group I: Served as a control group and were injected with isotonic saline for 7 days and then received a placebo for 2 weeks.
- Group II: Served as a positive control; injected with isoprenaline (isoproterenol) 5 mg/kg intraperitoneal injection for 7 days. Rats were then administered placebos for 2 weeks [9].

Group III, IV, and V group rats were injected with isoprenaline using the above mentioned method. Then:

- Group III: Received oral daily doses of sacubitril 30 mg/kg [10].
- Group IV: Received a combination of sacubitril 30 mg/kg/day with ramipril10mg/kg/day orally for 2 weeks [11].
- Group V: Received a combination of sacubitril 30 mg/kg/day with aliskiren 10 mg/kg/day orally for 2 weeks [12].

Medications were administered though oral gavage after dissolving them in water and calculating exact dose. Urine volume, blood pressure (blood pressure and heart rate were measured in the tails of the rats using a noninvasive CODA monitor system), body weight, and heart rate were monitored for 24 h and were recorded on the first day of the study in the Group I rats, on the eighth day of the experiment in Group II, and on the 21st day 3 h after the last doses of the drugs for the rest of the groups.

Urine was collected using a cylindrical cage; a funnel covered with mesh was attached to the bottom of the cage, to which a plastic container fitted for collecting urine.

On the 21st day of the experiment, all animals were anesthetized with intraperitoneal injection of Xylazine 10 mg/kg and Ketamine 125 mg/kg [13]. A cardiac puncture technique was used to collect blood; the samples were then centrifuged, and the separated sera were kept into tubes to be used for various tests.

The urine collected from different groups was used to estimate urine flow, total solute excretion, and glomerular filtration rate (GFR).

At the end of the experiment, while animals were anesthetized, animals were euthanized with cervical dislocation. Heartbeat and pupillary responses to light were assessed to confirm the deaths of the animals.

Serum and samples were coded with random numbers to blind the investigator to the samples and only after all samples have been assessed, the identity of each sample was revealed.

### Biochemical assays

Plasma NT-proBNP, renin, CK-MB, MMP 9, and troponin-I were measured using ELISA kits specific for rats. Urine and serum creatinine and blood urea nitrogen (BUN) were measured using specific reagents for each parameter mentioned through the use of the Cobas-Roche analyzer; urine sodium was measured using a flame photometer.

### Statistical analysis

All data were expressed as the mean ± standard error means (M ± SEM). The results were evaluated by using the SPSS (Version 23) computer program, and the differences in all parameters control and medication-treated rats were analyzed by one-way analysis of variables (ANOVA). The comparison between groups was done using a Tukey test. A change was considered statistically significant when *P* < 0.05.

## Results

### Effects of different treatment groups on biochemical assays

Results addressed in Table 1 confirm that the combination of sacubitil-ramipril and sacubitil-aliskiren significantly reduced the level of troponin-I (2.1 ± 0.16 ng/ml and 1.78 ± 0.2 ng/ml) respectively when compared to the troponin level in Group II (3.09 ± 0.147 ng/ml). However, the reduction is not significant when it comes to the control group (0.62 ± 0.12 ng/ml); sacubitril alone was not able to reduce troponin significantly compared to Groups I and II. None of the treatment groups were able to return the troponin-I level back to normal, and the difference with the control group is significant in all treatment groups.

**Table 1:** Effects of Sacubitril, Sacubitril-Aliskiren and Sacubitril-Ramipril on cardiac and renal biomarkers: troponin I, NT-proBNP, CK-MB, MMP9 and renin

Parameter	Control	Heart failure	Sacubitril	Ramipril & sacubitril	Aliskiren & sacubitril
troponin I ng/ml	0.62 ± 0.12	3.09 ± 0.147^**^	2.32 ± .0.28	2.1 ± 0.16^*^	1.78 ± 0.201^*^
NT-proBNP pg/ml	356 ± 27.61	980 ± 116.71^**^	658 ± 58.68^*^	471.66 ± 43.58^*^	462 ± 35.52^*^
CK-MB ng/ml	13.78 ± 1.67	31.55 ± 1.69^**^	28.71 ± 3.12	17 ± 3.7^*^	22.18 ± 1.63
MMP9 ng/ml	9.91 ± 0.43	15.85 ± 0.57^**^	12.45 ± 1.1^*^	11.99 ± 0.93^*^	11.3 ± 0.73^*^
renin pg/ml	408 ± 11.456	736 ± 45.8	693 ± 42.35	830.33 ± 23.35	836 ± 0.73^*^

Values are expressed as mean ± SEM

^*^P < 0.05 when compared with group II

^**^P < 0.05 when compared with group I

Results showed that Group II rats have significantly higher NT-proBNP plasma levels (980 ± 116 pg/ml) than the control group (356 ± 27 pg/ml) (*P* < 0.05). The NT-proBNP level was significantly reduced in all treatment groups (*P* < 0.05), and only Group IV and V rats showed no significant difference from the control group (*P* > 0.05).

The intraperitoneal injection of isoprenaline in Group II rats significantly raised the level of CK-MB (31.55 ± 1.69 ng/ml) when compared to Group I (13.78 ± 1.67 ng/ml) (*P* < 0.05). With the exception of the sacubitril-ramipril combination, CK-MB level was not reduced significantly by sacubitril alone or with its combination with aliskiren (*P* > 0.05).

Heart-failure induction by isoprenaline resulted in an increase in the circulating MMP9 (15.85 ± 0.57 ng/ml), and the increase was significant statistically (*P* > 0.05) when compared to the control group (9.91 ± 0.43 ng/ml). The results in Table 1 confirm that all treatment groups had significantly reduced MMP9 levels when compared to rats with induced heart failure (*P* < 0.05), and the MMP9 levels returned to normal.

Renin-plasma concentration in isoprenalinepretreated rats was significantly higher (736 ± 45.8 ng/ml) than that of control group (408 ± 11.456 ng/ml) (*P* < 0.05). However, rats treated with sacubitril-ramipril or sacubitril-aliskiren combinations showed higher plasma renin than sacubitril-treated rats. Rats treated with the sacubitril-aliskiren combination showed the highest renin levels when compared to rats in the other treatment groups.

### Effects of different treatment groups on renal function

The glomerular filtration rate was significantly reduced in rats injected with high doses of isoprenaline (7.031 ± 1.6 ml/h/kg) when compared to the control (140.5 ± 14.7 ml/h/kg), as shown in Table 2. All treatment groups presented an increase in GFR, and the increment was statistically significant compared to Group II (*P* < 0.05). Rats treated with the sacubitril-aliskiren combination established the highest GFR level (270.24 ± 73.31 ml/h/kg) compared to rats in other treatment groups. Compared to the control group, serum creatinine and BNU levels were significantly increased (*P* < 0.05) in rats with experimentally induced heart failure, and BUN level was reduced in all treatment groups compared to Group II, but statistical analysis showed that the difference was not significant with Group I (*P* > 0.05). However, only the sacubitril-aliskiren combination (35.93 ± 7.8 mg/dl) among all treatment groups showed no significant difference from the control group (32.5 ± 1.05 mg/dl). The results in Table 2 confirm that all treatment regimens were able to significantly increase urine flow. It was significantly reduced in group II (*P* < 0.05), while no statistically significant difference appeared between different treatment groups; similarly, the total solute-excretion value was increased significantly in all treatment groups. Again, the aliskiren-sacubitril combination presented the highest increase in total solute-excretion value (*P* < 0.05).

**Table 2:** Effects of Sacubitril, Sacubitril-Aliskiren and Sacubitril-Ramipril on renal function: GFR, creatinine, urine flow and total solute excretion

Parameter	Control	Heart failure	Sacubitril	Ramipril & sacubitril	Aliskiren & sacubitril
GFR ml/h/kg	140.5 ± 14.7	7.031 ± 1.6	248.76 ± 44.91^*^	213.30 ± 29.81^*^	270.24 ± 73.31^*^
BUN mg/dl	32.5 ± 1.05	52.1 ± 1.57^**^	47.2 ± 2.13	49.3 ± 3.35	35.93 ± 7.8
Creatinine mg/dl	0.37 ± 0.02	0.92 ± 0.04^**^	0.498 ± 0.02^*^	0.67 ± 0.02^*^	0.66 ± 0.04^*^
Urine flow ml/h/kg	0.525 ± 0.09	0.2761 ± 0.06	1.0607 ± 0.23^*^	0.9593 ± 0.15^*^	1.0776 ± 0.23^*^
Total solute excretion meq/minute	0.594 ± 0.06	0.11 ± 0.03	0.912 ± 0.235^*^	0.765 ± 0.11	1.01 ± 0.29^*^

Values are expressed as mean ± SEM

^*^P < 0.05 when compared with group II

^**^P < 0.05 when compared with group I

### Effects of different treatment groups on blood pressure and heart rate

The results in Table 3 show that mean blood pressure and systolic blood pressure in all groups were significantly lower than in Groups I and II (*P* < 0.05), except for the sacubitril-ramipril combination, which did not show significant differences on either occasions (*P* > 0.05). Diastolic blood pressure was significantly reduced in all groups compared to the control group (*P* < 0.05), and only the sacubitril-aliskiren group had significant differences compared to Group II (*P* < 0.05).

**Table 3:** Effects of Sacubitril, Sacubitril-Aliskiren and Sacubitril-Ramipril on arterial blood pressure and heart rate: systolic blood pressure, diastolic blood pressure, mean blood pressure, and heart rate

Parameter	Control	Heart failure	Sacubitril	Ramipril & sacubitril	Aliskiren & sacubitril
Systolic blood pressure mm Hg	116.16 ± 1.6	104.16 ± 3.45^**^	91 ± 1.34^*^	95 ± 3.14	84.66 ± 2.96^*^
Diastolic blood pressure mm Hg	85.5 ± 0.56	72.5 ± 1.8^**^	66.3 ± 0.71	75.3 ± 3.6	65.3 ± 3.1
Mean blood pressure mm Hg	97.3 ± 0.76	83.5 ± 2.6^**^	74.3 ± 0.8^*^	80.16 ± 2.3	71.5 ± 2.94^*^
Heart rate beat/minutes	355 ± 18.2	505.8 ± 15.15^**^	427 ± 8.56^*^	368.8 ± 12.7^*^	400.16 ± 25.8^*^

Values are expressed as mean ± SEM

^*^P < 0.05 when compared with group II

^**^P < 0.05 when compared with group I

Heart rates were significantly higher in rats with heart failure when compared to the control rats (*P* < 0.05). Rats with heart failure expressed significantly higher heart rates than control rats. The sacubitril group rats did not experience any significant difference from Group II rats; however, both combinations were able to improve the heart rate in rats.

## Discussion

From the results shown in Table 3, it is very clear that intraperitoneal injection of isoprenaline 5 mg/kg/day for 7 days established physiological changes similar to heart failure in the rats. Triggering RAAS plays a critical role in the development of hemodynamic changes and cardiac remodeling; this model has been proven to successfully establish heart failure in rats [14].

Mechanisms proposed to explain the inductive effects of isoprenaline in heart failure in rats include distorted balance between oxygen demand and supply from cardiac myocytes, due to the extreme increase of heart rate and force of myocardial contraction [15].

Among all troponin subtypes, troponin-I appears to be the most sensitive indicative measure in myocardial infarction. In the normal physiological action of the heart, plasma the level of troponin-I appears to be zero or very low. In this study, the plasma troponin-I level significantly increased after heart-failure induction in Groups III, IV, and V. This rise in troponin plasma level is due to myocardial injury, and troponins are released as a result of this damage. This finding is in agreement with results from other studies [16]. The persistence of increased levels after 2 weeks of treatment means that the damage was not reversible, and the physiological modifications that took place with the assistance of therapy were of slight value in this regard. However, combinations of treatments were able to reduce the troponin-I value significantly when compared to Group II, indicating an alleviating effect of these two combinations in this aspect. Physiological changes triggered by sacubitril alone failed to show statistical decrements of troponin-Ivalue compared with Group II.

Both NT-proBNP and BNP are metabolic products of proBNP that is secreted into blood during myocardial stress, and are used as important monitoring tools in the pharmacotherapy of patients suffering from various pathological heart conditions [17]. NT-proBNP was used in this study rather than the BNP itself for two reasons. First, NT-proBNP has a longer half-life compared to BNP; second, studies have shown that the NT-proBNP value is not affected by neprilysin-inhibitor treatment—unlike BNP, which shows variations in patients on neprilysin-inhibitor therapy [18].

Since the NT-proBNP level is related to the stress on ventricular walls, results of this study show that combinations of sacubitril with ramipril or aliskiren significantly reduced the workload on the ventricular wall at a higher degree than sacubitril alone in rats with heart failure, as both Groups IV and V showed nonsignificant differences with control group. This is probably due to a further reduction in cardiac workload for their inhibition of RAAS and the consequent reduction of peripheral vascular resistance.

CK-MB appears to be the most specific isoform of creatinine kinases related to cardiac-muscle damage. Similar to NT-proBNP, CK-MB is also used as a diagnostic tool to evaluate the degree of myocardial damage [19]. The reduction of CK-MB values in Groups IV and V indicates attenuation of the process of cardiac injury. It also indicates that sacubitril alone has failed to improve this condition. The ramipril-sacubitril group appears to be the most effective combination in attenuating the process of cardiac injury.

Matrix metalloproteinases are zinc-dependent peptidases that break extracellular matrix proteins. Like other physiological peptides and enzymes, it has many isoforms. The isoform MMP9 is used as an efficient biomarker for cardiac remodeling [20]. Studies have concluded that increased plasma MMP9 levels are associated with increased left-ventricular diastolic dimensions and increased wall thickness. Furthermore, increased levels of MMP9 are linked with extended left-ventricular enlargement [21, 22]. Results of this study indicate that all treatments used were able to attenuate the process of cardiac remodeling.

An increase of renin level is expected in heart failure. The plasma level of the renin is increased in patients with heart failure as a result of neurohumoral changes that are activated in heart failure; this usually leads to negative consequences in overall health conditions [23]. In this study, the combination of aliskiren-sacubitril reduced plasma renin activity despite the increase in plasma-renin concentration, since aliskiren blocks the active site of renin, hence reducing its plasma activity. However, this will result in an increase in plasma renin levels because this will activate the negative feedback loop of the RAAS, which in turn leads to an increase in plasma-renin concentration [24].

All of the parameters mentioned above indicate the reduction of work stress on the heart in rats treated with medication. This decline appears in the form of reduced troponin-I, MMP9, and NT-proBNP, which are released upon myocardial injury, cardiac remodeling, and ventricular-wall stress, respectively. The increased renin-plasma concentration also reflects the feedback mechanism for RAAS inhibition and represents the efficiency of these medications to reduce the impact of neurohumoral changes on kidneys.

Inducing heart failure with isoprenaline showed a significant reduction in GFR and urine flow of rats. This is probably because physiological adaptations in heart failure are not enough to compensate for the failing heart [25]. Additionally, the kidneys will not be able to efficiently excrete urea and creatinine, which will lead to an increase in BUN and creatinine, collectively indicating compromises in overall renal function [26].

Sacubitril inhibits neprilysin that is responsible for degradation of natriuretic peptides; hence, natriuretic peptides enhance natriuresis and participate in the process of increasing urine flow, GFR, urea, and creatinine clearance. These activities may attenuate the increased blood levels of urea and creatinine. Adding aliskiren or ramipril to sacubitril will further improve renal function through interfering with RAAS and inhibiting production of the vasoconstrictor angiotensin II, along with the possible subsequent reduction in aldosterone secretion. This is clearly apparent in the results of total solute excretion, which increased significantly in all treatment groups compared to Group II.

Along with the inability of heart to pump blood efficiently due to compromised heart function induced with isoprenaline, a neprilysin inhibitor alone or in combination with ramipril or aliskiren stimulates natriuresis, which will consequently lead to further drops in the arterial blood pressure. Heart rate was reduced in all treatment groups, and heart rate in rats treated with the aliskiren-sacubitril combination showed no significant difference with rats in the control group.

Although the combination of both aliskiren with sacubitril or with ramipril appears to be effective in modulating neurohumoral changes associated with heart failure, results conclude that the aliskiren-sacubitril combination is more efficient than the ramipril combination. These results indicate that a complete suppression of RAAS with aliskiren provides better relief than blocking angiotensin II production with ramipril [27].

## Conclusion

sacubitril in combination with aliskiren or with ramipril have effectively reduced plasma-cardiac biomarkers such as CK-MB, MMP9, and NT-proBNP in rats with heart failure. Both combinations showed significant remediation of renal function through increasing GFR, urine flow, and total solute excretion, as well as reducing plasma levels of renin. Net results revealed that the sacubitril-aliskiren combination had similar remediating effects on neurohumoral changes compared to the sacubitril-ramipril combination.

## Abbreviations

ANP: Atrial natriuretic peptide
BNP: Brain natriuretic peptide
BUN: Blood urea nitrogen
CK-MB: Creatine kinase-MB
CNP: C-Type natriuretic peptide
ELISA: Enzyme Linked Immunosorbent Assay
GFR: Glomerular filtration rate
H: Hour
MMP9: Matrix metalloproteinase 9
NPs: Natriuretic peptides
NT-proBNP: N-terminal pro B-type natriuretic peptide
RAAS: renin-angiotensin-aldosterone system
